# Assessment of Status of *rpoB* Gene in FNAC Samples of Tuberculous Lymphadenitis by Real-Time PCR

**DOI:** 10.1155/2012/834836

**Published:** 2012-08-15

**Authors:** Amita Raoot, Geeta Dev

**Affiliations:** ^1^Department of Pathology, University College of Medical Sciences, Shahdara, Delhi 110095, India; ^2^Directorate of Health Services, Government of NCT of Delhi, Karkardoma, Delhi 110032, India

## Abstract

*Introduction.* Multidrug resistance tuberculosis (MDR TB), the combined resistance of *Mycobacterium tuberculosis* to isoniazid (INH) and rifampin (RFM) is a major public health problem in India as it ranks second among the MDR-TB high burden countries worldwide. WHO recommends RFM resistance as a “surrogate marker” for detecting MDR. FNAC is the most widely used noninvasive investigative technique for TB lymphadenitis. Real-time polymerase chain reaction, an extremely versatile technique can be used for the timely detection and treatment of MDR TB by assessing RFM resistance status in the FNAC samples of TB lymphadenitis. *Aim.* To assess the status of *rpoB* gene by real-time PCR in FNAC samples of TB lymphadenitis. *Materials and Methods.* Thirty FNAC samples from patients with persistent LAP or appearance of new LAP after 5 months or more of Anti Tubercular Treatment were assessed for status of *rpoB* gene by Real-Time PCR using probe covering the “hot spot resistance” region of the *rpoB* gene. *Result.* By using probe covering codons 531 and 526 of *rpoB* gene, we could detect 17 of 30 (56.7%) rifampin resistant isolate. The PCR could detect *Mtb* DNA in 100% of cases. *Conclusion.* Use of molecular methods like Real-Time PCR for detection of MDR-TB in FNAC samples is time saving, logical and economical approach over the culture based method.

## 1. Introduction

 Multidrug resistant tuberculosis (MDR-TB) is a major public health problem in India as it ranks second among the MDR-TB high-burden countries worldwide [1]. MDR-TB is defined as the combined resistance of *Mycobacterium tuberculosis *(*Mtb*) to isoniazid (INH) and rifampin (RFM). However, resistance to RFM, the first-line antituberculosis drug is considered to be more critical since it usually occurs in combination with other drugs specially INH. Hence, WHO has recommended RFM resistance as a “surrogate marker” for detecting MDR [2]. Ninety-six percent of RFM-resistant *Mtb *strains possess genetic alterations within an 81 bp “rifampin resistance-determining region” (RRDR) in the *rpoB* gene [3, 4], corresponding to codons 507 to 533. Real-time polymerase chain reaction (PCR) is a rapid and reliable method that enables both the amplification and the detection of mutations by using fluorescently labelled DNA probes [5]. The assessment of RFM resistance in cases of TB lymphadenitis (LAP), the most frequent (30–52%) cause of LAP in developing countries [6], is important for the timely detection and efficient treatment of such cases. Fine needle aspiration cytology (FNAC), a widely practised noninvasive, safe, simple, and rapid method of investigation of LAP, can be combined with rapid reliable technique like real-time PCR for early diagnosis and efficient management of MDR cases.

 In the present study, status of *rpoB* gene was assessed by real-time PCR in FNAC samples from 30 patients with persistent LAP or appearance of new LAP after 5 months or more of antitubercular treatment (“failures” as per Drug Resistance in World, WHO Global Report no. 3 2004). 

## 2. Aim of the Study

The aim of the study is to detect the status of *rpoB* gene by real-time PCR on FNAC samples from failures of TB LAP. 

## 3. Materials and Methods

### 3.1. Patients and Specimens

The present study was conducted prospectively over a period of one year on patients undergoing standardised ATT under the directly observed short course treatment (DOTS) regimen in Guru Teg Bahadur Hospital, a major tertiary health-care centre in East Delhi, India. Thirty clinico-cytologically confirmed cases of TB LAP who had taken 5 months or more of ATT and showed persistence of initial LAP or appearance of new LAP (failures [2]) were included in the study. Ten cases of non-TB LAP consisting of 4 lymphomas, 3 metastatic carcinomas with necrosis (histologically proven), and 3 cases of pyogenic abscess (gram stain positive for cocci) were included as negative controls. Relevant clinical history and examination findings were recorded. The FNAC material obtained was used for cytological diagnosis (Giemsa smears), demonstration of* Mycobacterium tuberculosis *(Ziehl Neelsen method), culture (Lowenstein-Jensen slants), and molecular diagnosis.

**DNA Extraction**

*Mycobacterial genomic* DNA was extracted as previously described by Van Sooligen et al. [7] with minor modifications. The quality and quantity of DNA were assessed by measuring absorbance ratio at 260/280 nm. An absorbance ratio within 1.8 to 2.0 suggested good quality extraction.

### 3.2. Real-Time PCR

All the 30 DNA samples were analysed by real-time PCR to assess the status of *rpoB* gene. A set of FRET probe that covered the most frequent mutation site in *rpoB* gene, codon region in 526 to 531 was used (Genosen's real-time PCR Kit, Genome Diagnostics Pvt. Ltd). This “resistance determining region” of *rpoB* gene was amplified, and the melting temperatures (*T*_*m*_s) of the probes were obtained on Rotor-gene 6000 real time rotary analyser (Corbett Research Australia) using FRET chemistry. It is an open chemistry platform that has 4–6 channel multiplexing capabilities and uses multiple excitation sources combined with several detection filters to detect virtually every known fluorophore. The change in the *T*_*m*_ was considered an indicator of a mutation, and isolates for which the probe had a *T*_*m*_ other than that for *M. tuberculosis *H37Rv was considered resistant to RFM [5]. The *T*_*m*_ of wild *rpoB* is 73.8–74.8°C and that of mutant *rpoB* is 69.8–70.8°C. A total of 25 microlitres of PCR mixture was prepared. It included ready-to-use reaction mixture containing the Genosens' *rpoB* real-time PCR kit, PCR buffer, hot start Taq polymerase, deoxynucleotide triphosphate, MgCl_2_, specific primers and probe. Five microlitres DNA of each isolate was then added. Real-time PCR was reformed in Rotor-gene 6000 real time rotary analyser (Corbett Research Australia). The cycling conditions were denaturing at 95°C for 10 min (Hold 1), followed by 45 cycles of amplification at 95°C for 15 secs, 58°C for 20 secs, 72°C for 20 secs. The conditions for the melt curve were as follows: Hold2 for 95°C, 1 min 0 secs, Hold3 @ 40°C, 0 min 30 secs, Hold4 @ 50°C, 0 min 30 secs. The melt started from 50°C to 90°C and the data was acquired on FAM/GREEN and CY5/RED channels. A positive control, negative control, and a nontemplate control (NTC) were also run. All the 10-negative controls were analysed similarly on real-time PCR.

### 3.3. Results

The age of the patients ranged from 11 to 50 years with a mean age of 24.20 years, and peak incidence was seen in the 2nd and 3rd decades of life. The male: female ratio was 0.76 : 1 with a slight female preponderance. Cervical region LAP was most common (54.5%). Multisite LAP was seen in 24% cases. The most common presenting complaint was fever with LAP in 48% patients, followed by asymptomatic LAP in 26.7%. BCG vaccination was recorded in 40% patients. Thin pus (described on the basis of the consistency of aspirated material) was seen in 47% followed by thick pus in 43.3% cases. The volume of FNA material varied from 0.5 mL to 2.5 mL. Necrosis with neutrophilic infiltrate and occasional epithelioid cell granulomas were the predominant cytomorphological pattern. Ninety percent cases were (AFBs) Acid-Fast Bacilli positive. *Mtb* could be isolated in culture in 6 (20%) cases.

### 3.4. Real-Time PCR Results

**Figure 1:**
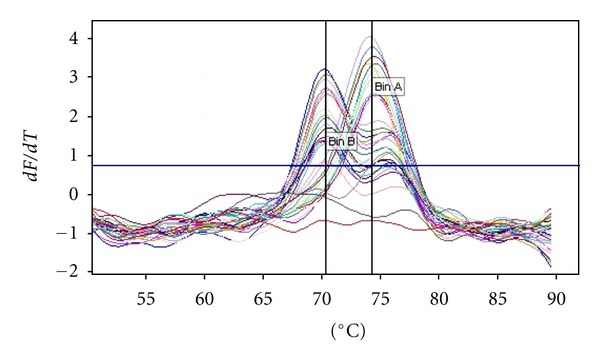
real time-PCR results showing two distinct peaks (*T*_*m*_) for wild and mutant *rpoB* gene. Melt data for melt A.green.

 Seventeen out of the 30 samples (56.7%) showed mutated *rpoB* gene and the remaining 13 (43.3%) had the wild *rpoB* gene (Table 1). We were able to detect 17 (56.7%) RFM-resistant isolates with the probe which covered codons 531 and 526 of the *rpoB* gene (Figure 1). The *T*_*m*_ of the probe for *Mtb *H37v was 73.8–74.8°C and that for the mutant *rpoB* probe was 69.8–70.8°C. The *T*_*m*_ for the RFM-resistant isolates created a drop of 4.0°C in the *T*_*m*_ of the probe.

## 4. Discussion

 The material obtained from FNAC, which has emerged as a safe, cost-effective first-line investigative technique in the evaluation of peripheral LAP, can be used for variety of clinical and ancillary tests like PCR [8, 9] that provide wide spectrum of information regarding diagnosis, treatment, and followup. Real-time PCR has been applied successfully in clinical samples directly [10, 11] for the detection of various microorganisms including *M. tuberculosis* [12]. Few studies in the literature have been published for direct detection of resistance in *Mtb* clinical samples using this method [10]. The main advantages of real-time PCR technique are rapid detection (1.5–2.0 hours after DNA extraction), quantitative analysis with the ability to pick up very minute quantity of DNA and a lower risk of contamination. The sensitivity has ranged from 71% to 98% with specificity close to 100% [13]. The method is of even greater importance in analysis of drug resistance in patient already on treatment as it is independent of presence of live bacilli which is crucial in all culture-based methods. The main handicaps are the requirement for expensive equipment and reagents, standardization of techniques, and the need for skilled technical personnel. Thus real-time PCR is time effective, sensitive, and specific method technique for detection of gene mutation in clinical samples.

 According to the WHO guidelines [2] MDR is defined as resistant to INH and RFM with or without resistance to other drugs. Rifampin is the most effective bactericidal drug against *Mtb*, binding with the DNA-dependent RNA polymerase to inhibit transcription and elongation of RNA [14, 15]. Out of the 4 subunits of this RNA polymerase, the *rpoB* gene-coded subunit is the most important because with genetic mutations in this unit, RFM is unable to bind with it [14] making the bacilli resistant to the drug. Resistance to RFM in *Mtb* occurs via genetic mechanism by random, single-step spontaneous mutation. Missense mutations at codons 526 to 531 have been reported to be crucial in conferring a high degree of resistance to RFM [16].

 The reported frequencies of mutations in *rpoB* are 41% in codon 531, 32–36% in codon 526, and 7–9% in codon 516 [4, 17]. In *Mtb* monoresistance to RFM is rare and at least 90% of all RFM-resistant clinical isolates are also resistant to INH. Hence, a positive result for RFM resistance is as useful as a surrogate of MDR-TB [2]. Thus use of molecular methods for detection of MDR in TB is a very logical, economical, and time-saving approach over the traditional non-molecular culture-based methods.

 In the present study, FNAC samples of TB LAP were studied for the status of *rpoB* gene by real-time PCR. By using *rpoB* probe that covered codons 531 and 526 of *rpoB* gene, we were able to detect 17 of 30 (56.7%) rifampin-resistant isolate. Earlier studies from South India have also shown that codons 531 and codon 526are most frequently involved in mutations [18, 19]. In the remaining 13 samples mutations could not be detected and hence they were labelled as wild. It is known that 3–5% of the RFM-resistant isolates do not possess any mutation in the *rpoB* gene [4]. The presence of additional mechanisms including RFM permeability and novel mutations in alternate subunits of RNA polymerase [16] are known to be involved in conferring the resistant phenotype. These mutations of the *rpoB* gene could have been missed in the study because we have used probe that detects mutation in the “hot spot” resistance conferring region of the *rpoB* gene.

 The PCR could detect *Mtb* DNA in 100% of cases whilst 90% were AFB positive by ZN, thus proving that real-time PCR is a more sensitive diagnostic technique for TB LAP over staining. We could detect DNA in two cases even when the sample was very small (less than 0.5 mL). It establishes the utility of real-time PCR in detecting smaller amount of DNA present in clinical samples that are difficult to obtain. 

 Most of the other studies [20, 21] carried out on FNAC samples of TB LAP have shown a culture positivity rate varying from 17–55.5%. In the present study, culture positivity was on lower side (20%). Low-culture positivity in this study could be because of several reasons, including presence of scant bacilli, presence of bactericidal substances as majority of the patients were on treatment, and harsh decontamination procedures [22]. The PCR positivity in culture-negative cases highlights the importance of this technique in detection of drug-resistant cases where culture based methods cannot be used. 

 With rise in MDR-TB, the early detection of RFM resistance in *Mtb*-infected patients is critically important not only for the patient management but also for the prevention of the spread of MDR strains, which if not controlled can have catastrophic effect on the TB-control programmes which hugely rely on RFM as the first-line drug of ATT. The standard methods for drug susceptibility testing (DST) of *Mtb* can take weeks to months to provide results. The combination of using a simple, safe, and rapid procedure like FNAC with a molecular technique like real-time PCR which is a fast, accurate, and sensitive will give an added advantage in reporting drug resistance in clinical isolates in a very short time.

**Limitation of Study**

We would like to mention that sequencing was not a part of our study. We were trying to assess the feasibility of utilizing relatively more accessible and simple techniques like real-time PCR which can be available in smaller setups. Sequencing is expensive and not easily available in smaller setups. In a developing country like ours easily accessible methods could be of greater importance for early effective management of patients. However sequencing could prove useful in the 13 cases where *rpoB* mutations could not be picked up by the probe used.

## Figures and Tables

**Table 1:** Real-time PCR results showing drop in *T*_*m*_. (wild/mutant *rpob* gene with different *T*_*m*_, Bin A: Wild, Bin B: Mutant).

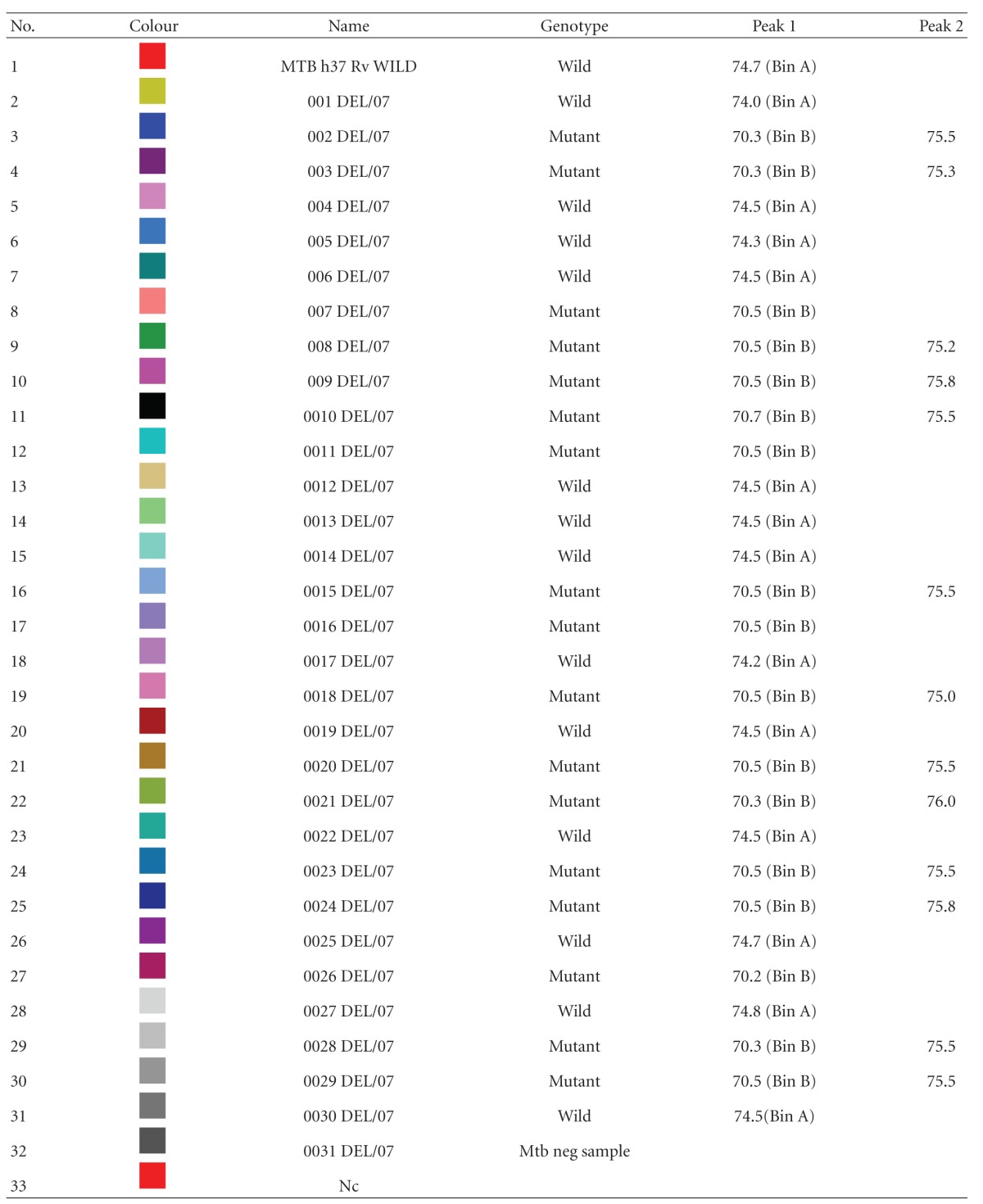

This report generated by Rotor-Gene 6000 Series Software 1.7 (Build 87) Copyright 2000–2006 Corbett Research, a Division of Corbett Life Science. All rights reserved. ISO 9001 : 2000 (Reg. no. QEC21313).
